# Targeting *Toxoplasma gondii* ME49 TgAPN2: A Bioinformatics Approach for Antiparasitic Drug Discovery

**DOI:** 10.3390/molecules28073186

**Published:** 2023-04-03

**Authors:** Ali Altharawi

**Affiliations:** Department of Pharmaceutical Chemistry, College of Pharmacy, Prince Sattam Bin Abdulaziz University, Al-Kharj 11942, Saudi Arabia; a.altharawi@psau.edu.sa

**Keywords:** *Toxoplasma gondii*, toxoplasmosis, computer-aided drug design, molecular dynamic simulation, MM-PBSA

## Abstract

As fewer therapeutic options are available for treating toxoplasmosis, newer antiparasitic drugs that can block TgAPN2 M1 aminopeptidase are of significant value. Herein, we employed several computer-aided drug-design approaches with the objective of identifying drug molecules from the Asinex library with stable conformation and binding energy scores. By a structure-based virtual screening process, three molecules—LAS_52160953, LAS_51177972, and LAS_52506311—were identified as promising candidates, with binding affinity scores of −8.6 kcal/mol, −8.5 kcal/mol, and −8.3 kcal/mol, respectively. The compounds produced balanced interacting networks of hydrophilic and hydrophobic interactions, vital for holding the compounds at the docked cavity and stable binding conformation. The docked compound complexes with TgAPN2 were further subjected to molecular dynamic simulations that revealed mean RMSD for the LAS_52160953 complex of 1.45 Å), LAS_51177972 complex 1.02 Å, and LAS_52506311 complex 1.087 Å. Another round of binding free energy validation by MM-GBSA/MM-PBSA was done to confirm docking and simulation findings. The analysis predicted average MM-GBSA value of <−36 kcal/mol and <−35 kcal/mol by MM-PBSA. The compounds were further classified as appropriate candidates to be used as drug-like molecules and showed favorable pharmacokinetics. The shortlisted compounds showed promising biological potency against the TgAPN2 enzyme and may be used in experimental validation. They may also serve as parent structures to design novel derivatives with enhanced biological potency.

## 1. Introduction

The M1 aminopeptidases (also termed aminopeptidase N) are found in living systems and are anchored in cell membrane [1]. These enzymes are vital from a functionality perspective and are important in cell growth, development, maintenance, and defense [2]. They are documented as potential candidates for novel drugs and regarded as potential immunoprotected targets [3]. In the *Plasmodium falciparum* malaria parasite, a single M1 aminopeptidase enzyme has been reported, classified as PfA-M1, that plays a prime role in hemoglobin digestion to ensure pathogen growth and development in the blood stage of the parasite [4]. Targeted inhibition of PfA-M1 has been unveiled to stop *P. falciparum* inhibition in both laboratory and animal murine models [5]. *Toxoplasma gondii* is a causative agent of toxoplasmosis, which can cause serious complications in pregnant women [2,6,7,8,9]. The congenital pathogen infection results in mental retardation, miscarriage, and hearing and vision problems [6]. Toxoplasmosis management relies on chemotherapy comprising pyrimethamine with either clindamycin or sulfadiazine [10]. Drugs that can treat both active and latent infections are scarce, and thus new biomolecular targets and their inhibitors are needed for drug discovery [11].

The M1 aminopeptidases are of three types in the *T. gondii* ME49 strain—TgAPN1, TgAPN2, and TgAPN3—and encoded by genes present on different chromosomes [2]. TgAPN1 is experimentally active and shown as immunogenic. TgAPN3 is a metalloexopeptidase enzyme and a functional peptide, despite the N-terminal transmembrane anchor. TgAPN2 has localization within the parasite cytosol and is expressed at all stages of the parasite life cycle. The biological function of TgAPN2 is still not clear; however, it is predicted to play a role in tachyzoite growth. Its upregulation in bradyzoites also makes it an important target for drug development [2].

In contrast to conventional drug discovery, computer-aided drug design (CADD) is considered attractive, as it is more cost-effective and consumes less time and little human resources [12,13,14]. CADD is a multidisciplinary approach and involves structure-based and ligand-based virtual screening of drug libraries against given biological targets [15,16]. The aim of the study was to identify the best binding molecules of *T. gondii* ME49 TgAPN2 that show stable binding mode associated with lowest binding energy score. The aim was split into several objectives. First, screening of the Asinex drug library was done by using blind screening to identify novel binding sites for inhibitors [17]. The docking analysis includes both identification of compound binding mode as well as binding affinity score [18,19]. As the docking and virtual screening calculations are associated with high false-positive rates, the study was extended by molecular dynamic simulation analysis, which aids in validating hit compound binding modes and interaction stability along the length of simulation time [20]. Molecular dynamic simulation also helps in pointing to protein regions that are more flexible and stable in the presence of compounds and residues that are vital in compound binding and biomolecule catalytic mechanisms. Dynamic simulation analysis is key in determining the binding and chemical interactions of lead molecules to the receptors and deciphering conformation stability during the simulation [21,22,23,24,25,26,27,28,29,30,31,32]. Binding free energy analysis was conducted to revalidate the docking and simulation findings [20,21]. Binding free energy methods rely on simulation trajectories and play a key role in compound structure optimization for lead discovery [25,26,27,28,29,30,31,32]. These methods have gained considerable appreciation in recent years, as they have proved vital in estimation of relative binding free energies [33,34]. Compared to molecular docking- or virtual screening-based approaches, these methods are more significant in predicting compound binding [35,36,37].The outcomes of this study may hasten antiparasitic drug development for controlling *T. gondii* ME49 diseases. Furthermore, new chemical structures may be designed from the compound’s parent structure in order to achieve good biological potency and a safer pharmacokinetic profile.

## 2. Results and Discussion

### 2.1. Structure-Based Virtual Screening

Virtual screening was conducted to identify compounds that show stable binding at the TgAPN2 binding pocket. This was achieved by screening the Asinex library, which contains diverse chemical scaffolds and ready-to use-products for experimental investigation. The virtual screening identified 10 compounds as promising leads (Table 1). Among these, only three were complexed with TgAPN2 for docking conformation and interaction analysis.

### 2.2. Top Three Compounds’ Docking Analysis

**Figure 1:**
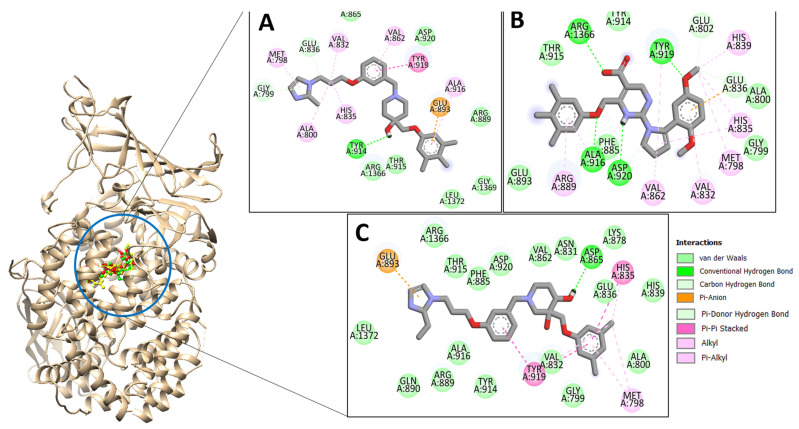
Docking conformation of compounds (shown by color ball and sticks) with TgAPN2 (shown by solid cartoon ribbon). (**A**) LAS_52160953, (**B**) LAS_51177972, and (**C**) LAS_52506311.

The top three compounds—LAS_52160953, LAS_51177972, and LAS_52506311—were chosen based on the lowest binding energy value in the virtual screening process, with binding energy values of −8.6 kcal/mol, −8.5 kcal/mol and −8.3 kcal/mol, respectively. Chemically, the LAS_52160953, LAS_51177972 and LAS_52506311 are 1-(3-(3-(2-methyl-2,3-dihydro-1H-imidazol-1-yl)propoxy)benzyl)-4-((3,4,5-trimethylphenoxy)methyl)piperidin-4-ol, 5-carboxy-2-(2-(2,5-dimethoxyphenyl)-1H-pyrrol-1-yl)-4-((3,4,5-trimethylphenoxy)methyl)pyrimidine-1-ium and 3-(phenoxymethyl)-1-(4-(2-(piperidin-1-yl)ethoxy)benzyl)piperidin-3-ol. All three compounds were noticed to achieve deep binding inside the active pocket and produced rich hydrophilic and hydrophobic contacts (Figure 1). The LAS_52160953 1-methyl-4-((3,4,5-trimethylphenoxy)methyl)piperidin-4-ol chemical moiety was seen docked deep inside the pocket facing the pocket bottom and formed a hydrogen bond contact with Tyr914 at distance of 2.3 Å. The opposite 2-methyl-1-(3-phenoxypropyl)-2,3-dihydro-1H-imidazole ring is placed at a pocket-out position and formed multiple weak hydrophobic contact. The compound interacts with several important residues, such as Asp920, Arg889, Gly1369, Leu1372, Thr915, Arg1366, Glu836 and Gly799. The LAS_51177972 was among the most stable compound due to multiple hydrogen bonds contact with the enzyme. The compound 5-methoxy-1,2,3-trimethylbenzene ring tend to interact with enzyme base residues while the opposite 5-carboxy-2-(2-(2,5-dimethoxyphenyl)-1H-pyrrol-1-yl)pyrimidine-1-ium interact with the active pocket walls. The compounds formed hydrogen bonds with Arg1366, Tyr919, Ala916 and Asp920 at distance of 2.3 Å, 3.1 Å, 2.6 Å, and 2.2 Å, respectively. LAS_52506311 3-(phenoxymethyl)piperidin-3-ol is involved in hydrogen bond contact Asp865 while the 1-(2-phenoxyethyl)piperidine ring interactions are dominated by van der Waals involving residues of Asp920, Val862, Gly836, Phe885, Thr915, Arg1366, Arg889, Ala916, and Leu1372 etc. The control compound 1 binding energy score was −8.52 kcal/mol. The control mainly formed hydrogen bonds with Asn831, His835 and Glu924. The van der Waals contacts were Val883, Arg879, Phe885, Gln866, Lys878, Leu886, Val832, Val862, and Tyr919 (Figure S1).

### 2.3. Molecular Dynamic Simulations

**Figure 2:**
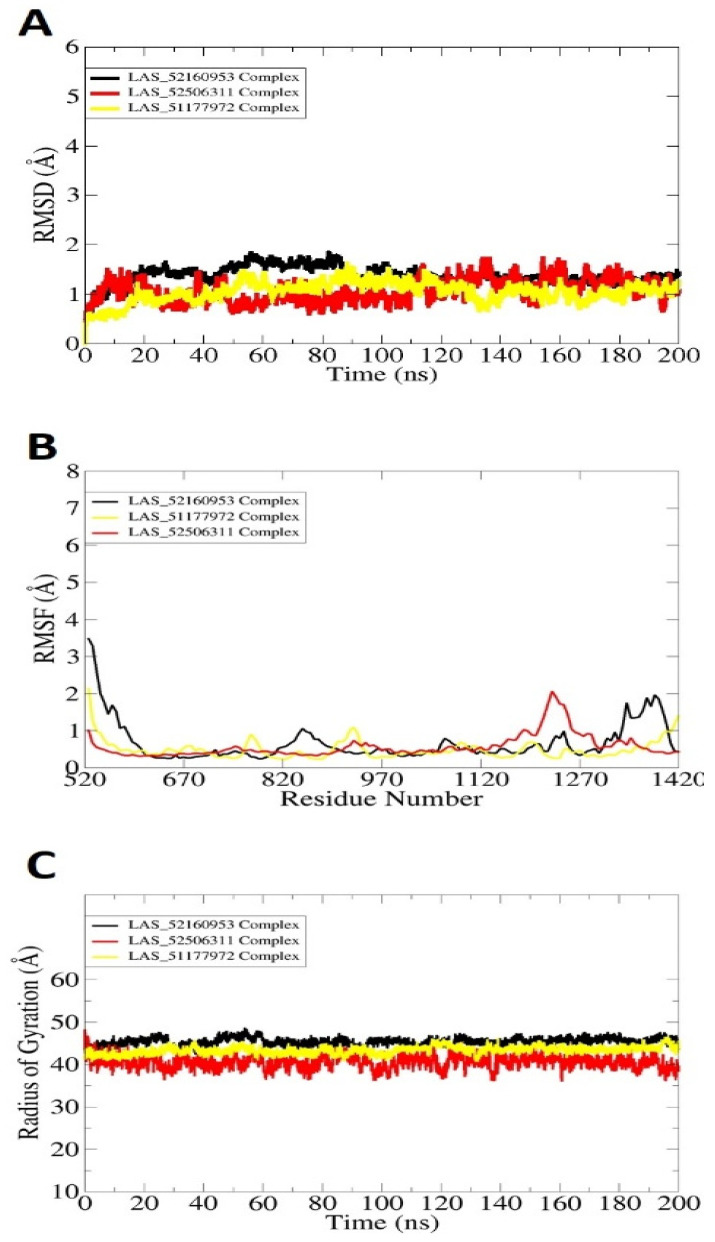
Molecular dynamic simulation analysis done using the CPPTRAJ module. (**A**) RMSD, (**B**) RMSF, and (**C**) RoG. All the analyses are plotted versus ångström.

Molecular dynamic simulations were conducted to investigate the physical movements of TgAPN2 in the presence of lead molecules. This helped in understanding the long-term compounds conformational stability with the enzyme and shed light on key molecular features of both receptor and compounds vital in intermolecular interactions. The analysis includes root mean square deviation (RMSD) [38,39], root mean square fluctuation (RMSF) [31,39] and radius of gyration (RoG) [4,5] (Figure 2). The analyses were done by mean of carbon alpha atoms. The RMSD describes the structure variation by measuring distance among the superimposed docked intermolecular conformation over the initial docked conformation. The RMSD demonstrated all the docked systems stable with mean RMSD value as LAS_52160953 complex (1.45 Å), LAS_51177972 complex (1.02 Å) and LAS_52506311 complex (1.087 Å). Among the systems, the first had more structural variations than the last two; however, it was still in a very stable range. All these values determine the complexes as highly stable from the docked conformation perspective. It further emphasizes that the compounds’ docked conformation with the enzyme is solid and no freedom of energy is available in the systems to detach the ligands. The control compound 1 RMSD is given in Figure S2, which shows mean RMSD of 2.4 Å. The reflects that the control docked complex with the enzyme showed more structure deviations than the filtered complexes. Previous studies reported that RMSD is important to determine structural stability of compounds at the receptor active pocket [39,40,41,42]. This was further validated by RMSF assay, which determines residue-based stability. RMSF analysis is important to underline the key regions of enzymes that play a vital role in compound recognition and long-term binding stability. RMSF, as can be seen in Figure 2B, evaluated that most of the receptor residues lie within the stable range in the presence of compounds. However, the N-terminal and C-terminal of the enzyme are a bit more flexible due to loops that are naturally flexible. The flexibility provides structure adaption to the enzyme during the catalytic process, thereby creating enough room for compounds to attain stable energy. Next, the compact and relaxed 3D conformation of TgAPN2 was investigated using RoG [32,43,44]. This analysis is important to understand the compactness and relaxed nature of the enzyme in the presence of compounds during simulation. Higher RoG determines loose packing of the enzyme’s secondary structure elements which may results in ligand detachment. The RoG depicted that the systems are compact with no major variations seen. This analysis complements the RMSD in gaining confidence in the system’s intermolecular docked conformation. The RoG value of the system ranged between 43 Å and 45 Å. These values are within the stable range and depict the stable compound binding with the enzyme and higher complex intermolecular stability.

### 2.4. Binding Free Energy Calculations

The binding free energy calculation by MM-GBSA/MM-PBSA was carried out in order to reassess the docked affinity of compounds for *Toxoplasma gondii* ME49 TgAPN2. The details of binding free energies are given in Table 2. All the complexes revealed stable energies, with an average value <−36 kcal/mol. The overall energy was dominated by van der Waals forces, which illustrated the complexes were stabilized mainly by weak hydrophobic energy. The TgAPN2- LAS_52160953 complex, LAS_51177972 complex and LAS_52506311 complex had net MM-GBSA binding energy scores of −39.45 kcal/mol, −36.19 kcal/mol, and −39.4 kcal/mol, respectively. High contributions were seen from van der Waals energy: −36.89 kcal/mol for LAS_52160953 complex, −33.60 kcal/mol for LAS_51177972 complex, and −36.02 kcal/mol for LAS_52506311 complex. The electrostatic energy contribution, though, was less contributing than the van der Waals energy, but still played a favorable role in complex stabilization. There were high contributions for LAS_52160953 and LAS_52506311: −14.23 kcal/mol and −14.85 kcal/mol, respectively. The net gas phase energy involved insignificant energy contribution from internal energy. The solvation energy contribution for each complex in MM-GBSA was seen as unfavorable. Similarly, on MM-PBSA analysis, the complexes also revealed stable energies. The net energy MM-PBSA values for TgAPN2-LAS_52160953 complex, LAS_51177972 complex and LAS_52506311 complex were −38.57 kcal/mol, −35.23 kcal/mol and −37.12 kcal/mol, respectively. These energy values were more stable for complexes than reported by MM-GBSA. Binding entropy values of LAS_52160953, LAS_51177972 and LAS_52506311 were 16.14 kcal/mol, 17.31 kcal/mol and 16.78 kcal/mol, respectively.

### 2.5. WaterSwap Analysis

**Figure 3:**
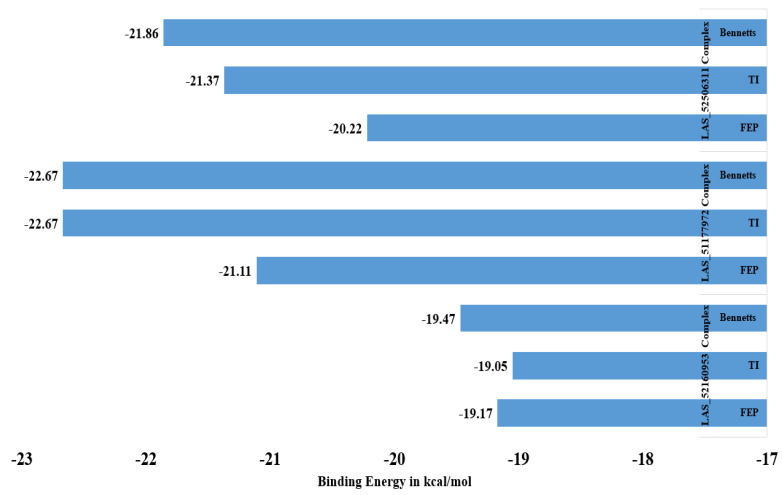
WaterSwap analysis energy values of complexes.

WaterSwap analysis was done to gain more confidence in the intermolecular affinity of complexes. The WaterSwap method is more sophisticated than the MM-GBSA/MM-PBSA method and considers the water molecules’ role in bridging ligand to receptor residues. This method has been successfully used in different studies and provided promising results in term of predicting compound binding affinity for the targeted biological macromolecule [27,45]. Three algorithms are used in WaterSwap: thermodynamic integration (TI), free energy perturbation (FEP), and Bennett’s. The three algorithms’ energy value for each complex is illustrated in Figure 3. All the three systems were confirmed to get maximum stable energy, depicting a strong interacting network.

### 2.6. SwissADME Analysis

In silico prediction of compounds ADMET properties is vital in present drug-discovery pipelines, as it saves cost and time associated with drug failure in clinical trials. The SwissADME details of LAS_52160953, LAS_51177972 and LAS_52506311 are given in Table 3. All the three compounds showed favorable adsorption, distribution, metabolism and excretion [46]. The compounds also revealed good topological polar surface area (TPSA) to allow efficient drug adsorption across the cell membrane [47]. Similarly, the compounds have good gastrointestinal absorption, and thus a high concentration of drugs can reach the site of action. Additionally, the compounds are drug-like, as classified by most rules, specifically Lipinski’s rule of five [48], Veber [49], and Egan [50]. Lipinski’s rule of five is a famous drug rule and describes that a biologically active drug molecule should possess several physical and chemical properties to be orally active. These properties include suitable molecular weight (<500 Da), hydrogen bond donors (<5), hydrogen bond acceptors (<10), and partition coefficient less than 5. Additionally, the compounds have no pan-assay interference compounds (PAINS) alerts and thus cannot bind to multiple targets and will specifically bind to a single biomolecule [51]. This prevents cross-reaction of the compounds with different proteins/enzymes when administered to the host and may also reduce unwanted and adverse effects. The compounds are synthetically feasible to synthesize. The easy synthesis of the compounds allows their use in experimental testing to disclose their real biological potency by blocking the biological function of the TgAPN enzyme.

## 3. Materials and Methods

### 3.1. Preparation of Protein Structure and Asinex Library

The crystallographic structure of *T. gondii* ME49 TgAPN2 was retrieved from the Protein Data Bank using PDB ID 6OIU [2,52]. The PDB structure was selected due to its recent submission to the database and good resolution quality compared to other available structures. The experimental data snapshot of the structure is as follows: method X-ray diffraction, resolution 2.20 Å, R-value free 0.236, R-value work 0.190, and R-value observed 0.193. The global stoichiometry of the structure is monomer. The native ligands were removed from the crystal structures along with water molecules. The missing amino acids were added using the CHARMM-GUI PDB manipulator [53]. The protonation of the structure was carried out at pH 7.4 using the H++ web server [54]. The structure was energy-minimized using steepest descent and conjugate gradient algorithms in UCSF Chimera v1.16 to eliminate steric clashes [55]. After energy optimization, the structure was saved in the PDB for additional analysis. The drug library used in this study was Asinex (https://www.asinex.com/ accessed on 15 November 2022). This library contains 575,302 compounds (updated on February 2023). It provides a cost-effective, diverse, drug-like chemical space. Most of the compounds in the library have a high degree of drug-likeness. The library was imported to PyRx 0.8 software [56]. The library was then energy-minimized using MM2 force field and converted to pdbqt format to make the compounds ready for virtual screening [57].

### 3.2. Virtual Screening

Virtual screening is a regularly used technique to screen drug libraries against any given enzyme/protein active pocket with the goal of identifying molecules that show best fitting with the receptor enzyme [58]. This technique has been used in different studies and provided lead molecules that produced good biological potency against the receptor [59]. The virtual screening process was initiated by docking all the Asinex library using the PyRx 0.8 AutoDock Vina program. The screening was performed against catalytic domain II by specifying the residues His835, His839 and Glu858 [2].The grid box dimensions were 25 Å on the XYZ axes. The grid box was centered on the X-axis = 90.81 Å, Y-axis = 25.40 Å, and Z-axis = 126.93 Å. The grid box in virtual screening was set as such to provide flexibility to the library compounds for binding the cavity across the *T. gondii* ME49 TgAPN2, where they show high conformational stability. The number of iterations set for each compound in docking was 100. The docking process was validated by redocking of cocrystalized ligand to the same site reported in the crystal structure. The superimposition of crystalized ligand and docked conformation revealed a root mean square deviation (RMSD) value of 0.27 Å, illustrating docking procedure accuracy [38]. For a positive control, compound 1 from Marijanovic et al., 2020 was used [2].

### 3.3. Molecular Dynamic Simulation

Molecular dynamic simulation is a computer-based approach to study physical movements of atoms/molecules in a fixed period of time [20]. The molecules’ movements are solved by Newton’s law of motion, and behavior is plotted in the form of a graph [60]. The top complexes (LAS_52160953, LAS_51177972 and LAS_52506311) were subjected to molecular dynamic simulations in explicit water. The starting atomic coordinates used were obtained from molecular docking and processed through the AMBER20 simulation package [61]. Amber ff14sb was employed for M1 aminopeptidase TgAPN2, while generalized Amber force field (GAFF) was applied for nonstandard residues as well as the TIP3P water model [62,63,64,65]. Amber ff14sb was used as it has improved potential for side chain torsion potentials. The compounds’ partial charges were added using the AM1-BCC method via antechamber program [66]. Each complex was placed at the center of a dodecahedral box with padding distance of 12 Å, followed by charge neutralization by adding an appropriate number of counterions. The number of sodium ions added were 12, 11 and 13 for LAS_52160953 complex, LAS_51177972 complex and LAS_52506311 complex, respectively. The complexes were next subjected to energy minimization for 5000 steps of the steepest descent algorithm in order to remove irregular geometry and steric clashes. During energy minimization, hydrogen atoms were minimized first for 500 cycles. This was followed by entire systems’ atom minimization with restraint of 10 kcal/mol Å^2^. Then, nonheavy atoms were lastly energy-minimized for 500 rounds in the presence of 200 kcal/mol Å^2^ on the remaining system. Afterward, equilibration was done for 100 ps under canonical ensemble. Then, another equilibration was performed for each system under isothermal isobaric ensemble in the presence of 1 bar pressure. Heating of each system was done gradually and achieved a constant temperature of 310 K. The production run was performed for 200 ns in periodic boundary conditions. The production run was facilitated in NVT (constant temperature, constant volume) ensemble and in the presence of Berendsen algorithm. The SHAKE algorithm and Langevin were considered for maintain restraint on hydrogen bonds and constant temperature, respectively [67,68]. The particle-mesh Ewald method was employed for treating long-range interactions. The cutoff value used for long-range interactions was 1.0 nm. The CPPTRAJ module of AMBER was used for structure stability analysis of systems [69]. Plots were generated using XMGRACE v 5.1 [70] and snapshots of simulation were visualized using VMD v 1.93 [71].

### 3.4. MM/PBSA Binding Free Energy Calculations

The docking programs offer simplified scoring functions for predicting ligand binding affinity for receptors. Therefore, they suffer from several limitations in accurate prediction of binding energy. Validation of the binding affinity of studied compounds was accomplished using molecular mechanics Poisson–Boltzmann surface area (MM/PBSA) and molecular mechanics generalized Born surface area (MM/GBSA) methods as implemented in AMBER MMPBSA.py [29,30,72,73,74]. In the process, 1000 snapshots were extracted from the simulation trajectories. The MM/PBSA net binding energy was determined using the following equation:

Δ*G*bind = *G*complex − (*G*receptor + *G*ligand)

During the analysis, the nonpolar energies were estimated using linear combination of pairwise overlaps with water probe radius of 1.4 Å. The β and γ values of nonpolar solvation energy employed were 0 kcal/mol in MMGBSA and 0.092 kcal/mol in MMPBSA. The dielectric constant value in MM-GBSA was set to 1.0. The entropy energy contribution in complex formation was determined using a bash script described by Duan et al., 2016 [75]. The MM-GBSA and MM-PBSA binding energies were reconfirmed by WaterSwap, which is considered more sophisticated in terms of determining water molecules’ contribution in ligand binding to the receptor [76,77].

### 3.5. Computational Pharmacokinetic Analysis

Computational studies on compounds’ physicochemical properties, lipophilicity, water solubility, pharmacokinetics, drug-likeness, and medicinal chemistry were done using SwissADME webserver [78].

## 4. Conclusions

In this work, three high-affinity binders were identified—LAS_52160953, LAS_51177972, and LAS_52506311—against TgAPN2. The molecules achieved stable binding at the enzyme active pocket and produced a stable interacting network of hydrophilic and hydrophobic contacts. The docked complexes also demonstrated stable dynamics with no major structure variations. The compound binding of compounds was confirmed by different binding free energy methods. The binding energy of intermolecular complexes was dominated by van der Waals energy. Additionally, the electrostatic energy had a vital contribution in making the compounds’ stable binding mode. Though results of the study are promising, revalidation of biological potency can be ensured by subjecting the compounds to in vitro and in vivo studies. Further, the lead compounds’ structures might be used in designing novel promising derivatives that have fewer structure and physicochemical limitations.

## Figures and Tables

**Table 1:** Compounds identified via virtual screening that showed stable binding affinity for TgAPN2 binding pocket.

Ligand	Structure	IUPAC Name	Binding Affinity
LAS_52160953	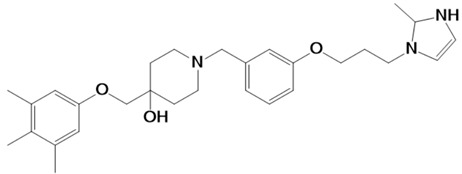	1-(3-(3-(2-methyl-2,3-dihydro-1H-imidazol-1-yl)propoxy)benzyl)-4-((3,4,5-trimethylphenoxy)methyl)piperidin-4-ol	−8.6
LAS_51177972	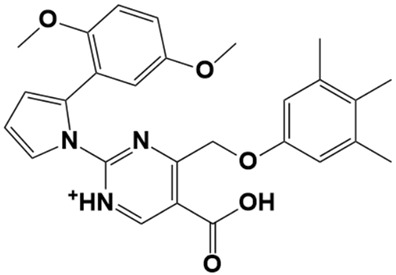	5-carboxy-2-(2-(2,5-dimethoxyphenyl)-1H-pyrrol-1-yl)-4-((3,4,5-trimethylphenoxy)methyl)pyrimidin-1-ium	−8.5
LAS_52506311	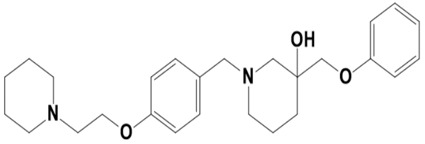	3-(phenoxymethyl)-1-(4-(2-(piperidin-1-yl)ethoxy)benzyl)piperidin-3-ol	−8.3
LAS_52160943	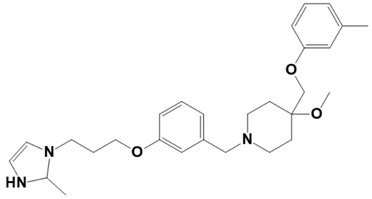	4-methoxy-1-(3-(3-(2-methyl-2,3-dihydro-1H-imidazol-1-yl)propoxy)benzyl)-4-((m-tolyloxy)methyl)piperidine	−7.8
LAS_52157607	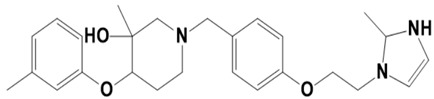	3-methyl-1-(4-(2-(2-methyl-2,3-dihydro-1H-imidazol-1-yl)ethoxy)benzyl)-4-(m-tolyloxy)piperidin-3-ol	−7.7
LAS_52160863	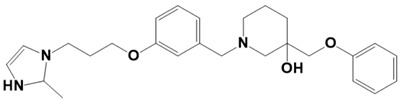	1-(3-(3-(2-methyl-2,3-dihydro-1H-imidazol-1-yl)propoxy)benzyl)-3-(phenoxymethyl)piperidin-3-ol	−7.7
LAS_52171211	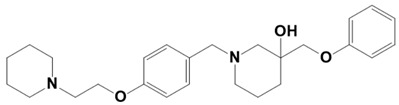	3-(phenoxymethyl)-1-(4-(2-(piperidin-1-yl)ethoxy)benzyl)piperidin-3-ol	−7.6
LAS_52506188	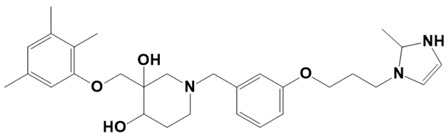	1-(3-(3-(2-methyl-2,3-dihydro-1H-imidazol-1-yl)propoxy)benzyl)-3-((2,3,5-trimethylphenoxy)methyl)piperidine-3,4-diol	−7.4
LAS_52157615	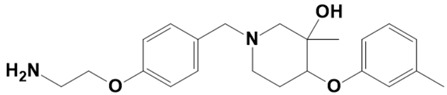	1-(4-(2-aminoethoxy)benzyl)-3-methyl-4-(m-tolyloxy)piperidin-3-ol	−7.1
LAS_32135590	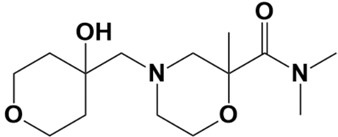	4-((4-hydroxytetrahydro-2H-pyran-4-yl)methyl)-N,N,2-trimethylmorpholine-2-carboxamide	−6.1

**Table 2:** MM-GBSA/MM-PBSA binding free energies of docked complexes, given in kcal/mol.

Energy Parameter	LAS_52160953 Complex	LAS_51177972 Complex	LAS_52506311 Complex
MM-GBSA			
van der Waals Energy	−36.89	−33.60	−36.02
Electrostatic Energy	−14.23	−12.78	−14.85
Delta Gas Phase Energy	−51.12	−46.38	−50.87
Delta Solvation Energy	11.67	10.19	11.47
Net Energy	−39.45	−36.19	−39.4
MM-PBSA			
van der Waals Energy	−36.89	−33.60	−36.02
Electrostatic Energy	−14.23	−12.78	−14.85
Delta Gas Phase Energy	−51.12	−46.38	−50.87
Delta Solvation Energy	12.55	11.15	13.75
Net Energy	−38.57	−35.23	−37.12

**Table 3:** SwissADME analysis of compounds. # stands for number.

Physicochemical Properties	LAS_52160953	LAS_51177972	LAS_52506311
Formula	C_29_H_41_N_3_O_3_	C_27_H_29_N3O_5_+	C_26_H_36_N_2_O_3_
Molecular weight	479.65 g/mol	475.54 g/mol	424.58 g/mol
Num. heavy atoms	35	35	31
Num. arom. heavy atoms	12	23	12
Fraction Csp3	0.52	0.22	0.54
Num. rotatable bonds	10	8	9
Num. H-bond acceptors	4	5	5
Num. H-bond donors	2	3	1
Molar Refractivity	153.33	134.40	132.19
TPSA	57.20 Å^2^	98.20 Å^2^	45.17 Å^2^
Lipophilicity			
Log *P*_o/w_ (iLOGP)	4.71	3.60	4.50
Log *P*_o/w_ (XLOGP3)	5.22	4.66	3.97
Log *P*_o/w_ (WLOGP)	3.21	3.84	3.04
Log *P*_o/w_ (MLOGP)	2.83	2.47	2.72
Log *P*_o/w_ (SILICOS-IT)	4.79	5.03	4.29
Consensus Log *P*_o/w_	4.15	3.92	3.70
Water Solubility			
Log *S* (ESOL)	−5.70	−5.68	−4.67
Solubility	9.66 × 10^−4^ mg/mL; 2.01 × 10^−6^ mol/L	9.88 × 10^−4^ mg/mL; 2.08 × 10^−6^ mol/L	9.16 × 10^−3^ mg/mL; 2.16 × 10^−5^ mol/L
Class	Moderately soluble	Moderately soluble	Moderately soluble
Log *S* (Ali)	−6.17	−6.45	−4.62
Solubility	3.25 × 10^−4^ mg/mL; 6.77 × 10^−7^ mol/L	1.69 × 10^−4^ mg/mL; 3.56 × 10^−7^ mol/L	1.02 × 10^−2^ mg/mL; 2.40 × 10^−5^ mol/L
Class	Poorly soluble	Poorly soluble	Moderately soluble
Log *S* (SILICOS-IT)	−7.47	−8.38	−6.78
Solubility	1.63 × 10^−5^ mg/mL; 3.39 × 10^−8^ mol/L	2.00 × 10^−6^ mg/mL; 4.21 × 10^−9^ mol/L	7.11 × 10^−5^ mg/mL; 1.67 × 10^−7^ mol/L
Class	Poorly soluble	Poorly soluble	Poorly soluble
Pharmacokinetics			
GI absorption	High	High	High
BBB permeant	Yes	No	Yes
P-gp substrate	Yes	No	Yes
CYP1A2 inhibitor	No	No	No
CYP2C19 inhibitor	No	Yes	No
CYP2C9 inhibitor	No	Yes	No
CYP2D6 inhibitor	Yes	Yes	Yes
CYP3A4 inhibitor	Yes	Yes	Yes
Log *K*_p_ (skin permeation)	−5.52 cm/s	−5.89 cm/s	−6.07 cm/s
Druglikeness			
Lipinski	Yes; 0 violation	Yes; 0 violation	Yes; 0 violation
Ghose	No; 2 violations: MR > 130, #atoms > 70	No; 1 violation: MR > 130	No; 1 violation: MR > 130
Veber	Yes	Yes	Yes
Egan	Yes	Yes	Yes
Muegge	No; 1 violation: XLOGP3 > 5	Yes	Yes
Bioavailability Score	0.55	0.55	0.55
Medicinal Chemistry			
PAINS	0 alert	0 alert	0 alert
Brenk	0 alert	No; 3 violations: MW > 350, Rotors > 7, XLOGP3 > 3.5	No; 3 violations: MW > 350, Rotors > 7, XLOGP3 > 3.5
Lead-likeness	No; 3 violations: MW > 350, Rotors > 7, XLOGP3 > 3.5	No; 1 violations: MW > 350	No; 1 violations: MW > 350
Synthetic accessibility	4.87	4.76	4.81

## Data Availability

The data presented in this study are available within the article.

## Supplementary Materials

The following supporting information can be downloaded at https://www.mdpi.com/article/10.3390/molecules28073186/s1, Figure S1: Docked conformation and interactions of control compound 1 with TgAPN2 (shown by secondary structure cartoon representation); Figure S2: RMSD of control compound 1.

## Sample Availability

Samples of the compounds are not available from the author.

## References

[1] Chen L., Lin Y.-L., Peng G., Li F. (2012). Structural basis for multifunctional roles of mammalian aminopeptidase N. Proc. Natl. Acad. Sci. USA.

[2] Marijanovic E.M., Swiderska K.W., Andersen J., Aschenbrenner J.C., Webb C.T., Drag M., Drinkwater N., McGowan S. (2020). X-ray crystal structure and specificity of the Toxoplasma gondii ME49 TgAPN2. Biochem. J..

[3] Drinkwater N., Lee J., Yang W., Malcolm T.R., McGowan S. (2017). M1 Aminopeptidases as Drug Targets: Broad Applications or Therapeutic Niche?. FEBS J..

[4] Mathew R., Wunderlich J., Thivierge K., Cwiklinski K., Dumont C., Tilley L., Rohrbach P., Dalton J.P. (2021). Biochemical and cellular characterisation of the Plasmodium falciparum M1 alanyl aminopeptidase (PfM1AAP) and M17 leucyl aminopeptidase (PfM17LAP). Sci. Rep..

[5] Bounaadja L., Schmitt M., Albrecht S., Mouray E., Tarnus C., Florent I. (2017). Selective inhibition of PfA-M1, over PfA-M17, by an amino-benzosuberone derivative blocks malaria parasites development in vitro and in vivo. Malar. J..

[6] Hill D., Dubey J. (2002). Toxoplasma gondii: Transmission, diagnosis and prevention. Clin. Microbiol. Infect..

[7] Liu Q., Wang Z.-D., Huang S.-Y., Zhu X.-Q. (2015). Diagnosis of toxoplasmosis and typing of Toxoplasma gondii. Parasites Vectors.

[8] Ali A., Naz A., Soares S.C., Bakhtiar M., Tiwari S., Hassan S.S., Hanan F., Ramos R., Pereira U., Barh D. (2015). Pan-Genome Analysis of Human Gastric Pathogen, H. Pylori: Comparative Genomics and Pathogenomics Approaches to Identify Regions Associated with Pathogenicity and Prediction of Potential Core Therapeutic Targets. Biomed. Res. Int..

[9] McGovern O.L., Rivera-Cuevas Y., Carruthers V.B. (2021). Emerging Mechanisms of Endocytosis in Toxoplasma gondii. Life.

[10] Milne G., Webster J.P., Walker M. (2020). Toxoplasma Gondii: An Underestimated Threat?. Trends Parasitol..

[11] Montazeri M., Mehrzadi S., Sharif M., Sarvi S., Tanzifi A., Aghayan S.A., Daryani A. (2018). Drug Resistance in Toxoplasma gondii. Front. Microbiol..

[12] Macalino S.J.Y., Gosu V., Hong S., Choi S. (2015). Role of computer-aided drug design in modern drug discovery. Arch. Pharm. Res..

[13] Van Drie J.H. (2007). Computer-aided drug design: The next 20 years. J. Comput. Aided Mol. Des..

[14] Yu W., MacKerell A.D. (2017). Computer-Aided Drug Design Methods. Antibiotics.

[15] Alamri M.A., Tariq M.H., Tahir ul Qamar M., Alabbas A.B., Alqahtani S.M., Ahmad S. (2023). Discovery of Potential Phytochemicals as Inhibitors of TcdB, a Major Virulence Factors of Clostridioides Difficile. J. Biomol. Struct. Dyn..

[16] Altharawi A., Riadi Y., Qamar M.T.U. (2023). An in Silico Quest for Next-Generation Antimalarial Drugs by Targeting Plasmodium Falciparum Hexose Transporter Protein: A Multi-Pronged Approach. J. Biomol. Struct. Dyn..

[17] Bechelane-Maia E.H., Assis L.C., Alves de Oliveira T., Marques da Silva A., Gutterres Taranto A. (2020). Structure-based virtual screening: From classical to artificial intelligence. Front. Chem..

[18] Meng X.-Y., Zhang H.-X., Mezei M., Cui M. (2011). Molecular Docking: A Powerful Approach for Structure-Based Drug Discovery. Curr. Comput. Aided-Drug Des..

[19] Huey R., Morris G.M. (2008). Using AutoDock 4 with AutoDocktools: A Tutorial. Scripps Res. Inst. USA.

[20] Hansson T., Oostenbrink C., van Gunsteren W. (2002). Molecular Dynamics Simulations. Curr. Opin. Struct. Biol..

[21] Qamar M.T.U., Ahmad S., Fatima I., Ahmad F., Shahid F., Naz A., Abbasi S.W., Khan A., Mirza M.U., Ashfaq U.A. (2021). Designing multi-epitope vaccine against Staphylococcus aureus by employing subtractive proteomics, reverse vaccinology and immuno-informatics approaches. Comput. Biol. Med..

[22] Ali S., Ali S., Javed S.O., Shoukat S., Ahmad S., Ali S.S., Hussain Z., Waseem M., Rizwan M., Suleman M. (2021). Proteome wide vaccine targets prioritization and designing of antigenic vaccine candidate to trigger the host immune response against the Mycoplasma genitalium infection. Microb. Pathog..

[23] Durrant J.D., McCammon J.A. (2011). Molecular dynamics simulations and drug discovery. BMC Biol..

[24] Ahmad S., Raza S., Uddin R., Azam S.S. (2018). Comparative subtractive proteomics based ranking for antibiotic targets against the dirtiest superbug: Acinetobacter baumannii. J. Mol. Graph. Model..

[25] Sanober G., Ahmad S., Azam S.S. (2017). Identification of plausible drug targets by investigating the druggable genome of MDR Staphylococcus epidermidis. Gene Rep..

[26] Muneer I., Ahmad S., Naz A., Abbasi S.W., Alblihy A., Aloliqi A.A., Alkhayl F.F., Alrumaihi F., Ahmad S., El Bakri Y. (2021). Discovery of Novel Inhibitors from Medicinal Plants for V-Domain Ig Suppressor of T-Cell Activation (VISTA). Front. Mol. Biosci..

[27] Ahmad S., Waheed Y., Ismail S., Abbasi S.W., Najmi M.H. (2020). A computational study to disclose potential drugs and vaccine ensemble for COVID-19 conundrum. J. Mol. Liq..

[28] Ehsan N., Ahmad S., Navid A., Azam S.S. (2018). Identification of potential antibiotic targets in the proteome of multi-drug resistant Proteus mirabilis. Meta Gene.

[29] Genheden S., Ryde U. (2015). The MM/PBSA and MM/GBSA methods to estimate ligand-binding affinities. Expert Opin. Drug Discov..

[30] Wang E., Sun H., Wang J., Wang Z., Liu H., Zhang J.Z.H., Hou T. (2019). End-Point Binding Free Energy Calculation with MM/PBSA and MM/GBSA: Strategies and Applications in Drug Design. Chem. Rev..

[31] El Bakri Y., Anouar E.H., Ahmad S., Nassar A.A., Taha M.L., Mague J.T., El Ghayati L., Essassi E.M. (2021). Synthesis and Identification of Novel Potential Molecules Against COVID-19 Main Protease Through Structure-Guided Virtual Screening Approach. Appl. Biochem. Biotechnol..

[32] Javed N., Ahmad S., Raza S., Azam S.S. (2021). Subtractive Proteomics Supported with Rational Drug Design Approach Revealed ZINC23121280 as a Potent Lead Inhibitory Molecule for Multi-Drug Resistant Francisella Tularensis: Drug Designing for Multidrug-Resistant Francisella Tularensis. Proc. Pak. Acad. Sci. B Life Environ. Sci..

[33] Alamri M.A., Ahmad S., Alqahtani S.M., Irfan M., Alabbas A.B., Qamar M.T.U. (2022). Screening of marine natural products for potential inhibitors targeting biotin biosynthesis pathway in Mycobacterium tuberculosis. J. Biomol. Struct. Dyn..

[34] Alamri M.A., Tahir ul Qamar M., Alabbas A.B., Alqahtani S.M., Alossaimi M.A., Azam S., Hashmi M.H., Rajoka M.S.R. (2022). Molecular and Structural Analysis of Specific Mutations from Saudi Isolates of SARS-CoV-2 RNA-Dependent RNA Polymerase and Their Implications on Protein Structure and Drug–Protein Binding. Molecules.

[35] Tahir ul Qamar M., Zhu X.-T., Chen L.-L., Alhussain L., Alshiekheid M.A., Theyab A., Algahtani M. (2022). Target-Specific Machine Learning Scoring Function Improved Structure-Based Virtual Screening Performance for SARS-CoV-2 Drugs Development. Int. J. Mol. Sci..

[36] Alamri M.A., Mirza M.U., Adeel M.M., Ashfaq U.A., Qamar M.T.U., Shahid F., Ahmad S., Alatawi E.A., Albalawi G.M., Allemailem K.S. (2022). Structural Elucidation of Rift Valley Fever Virus L Protein towards the Discovery of Its Potential Inhibitors. Pharmaceuticals.

[37] Ahmad F., Albutti A., Tariq M.H., Din G., Qamar M.T.U., Ahmad S. (2022). Discovery of Potential Antiviral Compounds against Hendra Virus by Targeting Its Receptor-Binding Protein (G) Using Computational Approaches. Molecules.

[38] Maiorov V.N., Crippen G.M. (1994). Significance of Root-Mean-Square Deviation in Comparing Three-dimensional Structures of Globular Proteins. J. Mol. Biol..

[39] Ahmad S., Raza S., Uddin R., Azam S.S. (2017). Binding mode analysis, dynamic simulation and binding free energy calculations of the MurF ligase from Acinetobacter baumannii. J. Mol. Graph. Model..

[40] Lobanov M.Y., Bogatyreva N.S., Galzitskaya O.V. (2008). Radius of Gyration as an Indicator of Protein Structure Compactness. Mol. Biol..

[41] Wahedi H.M., Ahmad S., Abbasi S.W. (2021). Stilbene-based natural compounds as promising drug candidates against COVID-19. J. Biomol. Struct. Dyn..

[42] Humayun F., Khan A., Ahmad S., Yuchen W., Wei G., Nizam-Uddin N., Hussain Z., Khan W., Zaman N., Rizwan M. (2021). Abrogation of SARS-CoV-2 Interaction with Host (NRP1) Neuropilin-1 Receptor through High-Affinity Marine Natural Compounds to Curtail the Infectivity: A Structural-Dynamics Data. Comput. Biol. Med..

[43] Khan S., Irfan M., Hameed A.R., Ullah A., Abideen S.A., Ahmad S., Haq M.U., El Bakri Y., Al-Harbi A.I., Ali M. (2022). Vaccinomics to design a multi-epitope-based vaccine against monkeypox virus using surface-associated proteins. J. Biomol. Struct. Dyn..

[44] Ahmad S., Ranaghan K.E., Azam S.S. (2019). Combating tigecycline resistant Acinetobacter baumannii: A leap forward towards multi-epitope based vaccine discovery. Eur. J. Pharm. Sci..

[45] Ain Q.U., Ahmad S., Azam S.S. (2018). Subtractive proteomics and immunoinformatics revealed novel B-cell derived T-cell epitopes against Yersinia enterocolitica: An etiological agent of Yersiniosis. Microb. Pathog..

[46] Van De Waterbeemd H., Gifford E. (2003). ADMET in Silico Modelling: Towards Prediction Paradise?. Nat. Rev. Drug Discov..

[47] Assenov Y., Ramírez F., Schelhorn S.-E., Lengauer T., Albrecht M. (2007). Computing topological parameters of biological networks. Bioinformatics.

[48] Lipinski C.A. (2004). Lead- and drug-like compounds: The rule-of-five revolution. Drug Discov. Today Technol..

[49] Veber D.F., Johnson S.R., Cheng H.-Y., Smith B.R., Ward K.W., Kopple K.D. (2002). Molecular Properties That Influence the Oral Bioavailability of Drug Candidates. J. Med. Chem..

[50] Egan W.J., Merz K.M., Baldwin J.J. (2000). Prediction of Drug Absorption Using Multivariate Statistics. J. Med. Chem..

[51] Whitty A. (2011). Growing PAINS in academic drug discovery. Futur. Med. Chem..

[52] Sussman J.L., Lin D., Jiang J., Manning N.O., Prilusky J., Ritter O., Abola E.E. (1998). Protein Data Bank (PDB): Database of Three-Dimensional Structural Information of Biological Macromolecules. Acta Crystallogr. Sect. D Biol. Crystallogr..

[53] Jo S., Cheng X., Islam M.S., Huang L., Rui H., Zhu A., Lee H.S., Qi Y., Han W., Vanommeslaeghe K. (2014). CHARMM-GUI PDB Manipulator for Advanced Modeling and Simulations of Proteins Containing Nonstandard Residues. Adv. Protein Chem. Struct. Biol..

[54] Anandakrishnan R., Aguilar B., Onufriev A.V. (2012). H++ 3.0: Automating pK prediction and the preparation of biomolecular structures for atomistic molecular modeling and simulations. Nucleic Acids Res..

[55] Kaliappan S., Bombay I.I.T. (2018). UCSF Chimera-Overview. http://doer.col.org/handle/123456789/9120.

[56] Dallakyan S., Olson A.J. (2015). Small-Molecule Library Screening by Docking with PyRx. Chemical Biology.

[57] Halgren T. (1996). A Merck Molecular Force Field. J. Comput. Chem..

[58] Horoiwa S., Yokoi T., Masumoto S., Minami S., Ishizuka C., Kishikawa H., Ozaki S., Kitsuda S., Nakagawa Y., Miyagawa H. (2019). Structure-based virtual screening for insect ecdysone receptor ligands using MM/PBSA. Bioorg. Med. Chem..

[59] Lionta E., Spyrou G., Vassilatis D.K., Cournia Z. (2014). Structure-Based Virtual Screening for Drug Discovery: Principles, Applications and Recent Advances. Curr. Top. Med. Chem..

[60] Shaker B., Ahmad S., Lee J., Jung C., Na D. (2021). In silico methods and tools for drug discovery. Comput. Biol. Med..

[61] Case D.A., Belfon K., Ben-Shalom I., Brozell S.R., Cerutti D., Cheatham T., Cruzeiro V.W.D., Darden T., Duke R.E., Giambasu G. (2020). Amber 2020, University of California, San Fransisco. J. Amer. Chem. Soc..

[62] Case D.A., Babin V., Berryman J.T., Betz R.M., Cai Q., Cerutti D.S., Cheatham T.E., Darden T.A., Duke R.E., Gohlke H. (2014). The FF14SB Force Field. Amber.

[63] Dickson C.J., Rosso L., Betz R.M., Walker R.C., Gould I.R. (2012). GAFFlipid: A General Amber Force Field for the accurate molecular dynamics simulation of phospholipid. Soft Matter.

[64] He X., Liu S., Lee T.-S., Ji B., Man V.H., York D.M., Wang J. (2020). Fast, Accurate, and Reliable Protocols for Routine Calculations of Protein–Ligand Binding Affinities in Drug Design Projects Using AMBER GPU-TI with ff14SB/GAFF. ACS Omega.

[65] Maier J.A., Martinez C., Kasavajhala K., Wickstrom L., Hauser K.E., Simmerling C. (2015). ff14SB: Improving the accuracy of protein side chain and backbone parameters from ff99SB. J. Chem. Theory Comput..

[66] Wang J., Wang W., Kollman P.A., Case D.A. (2001). Antechamber: An Accessory Software Package for Molecular Mechanical Calculations. J. Am. Chem. Soc..

[67] Kräutler V., Van Gunsteren W.F., Hünenberger P.H. (2001). A Fast SHAKE Algorithm to Solve Distance Constraint Equations for Small Molecules in Molecular Dynamics Simulations. J. Comput. Chem..

[68] Izaguirre J.A., Catarello D.P., Wozniak J.M., Skeel R.D. (2001). Langevin stabilization of molecular dynamics. J. Chem. Phys..

[69] Roe D.R., Cheatham III T.E. (2013). PTRAJ and CPPTRAJ: Software for Processing and Analysis of Molecular Dynamics Trajectory Data. J. Chem. Theory Comput..

[70] Turner P.J. (2005). XMGRACE, Version 5.1. 19.

[71] Humphrey W., Dalke A., Schulten K. (1996). VMD: Visual molecular dynamics. J. Mol. Graph..

[72] Genheden S., Kuhn O., Mikulskis P., Hoffmann D., Ryde U. (2012). The Normal-Mode Entropy in the MM/GBSA Method: Effect of System Truncation, Buffer Region, and Dielectric Constant. J. Chem. Inf. Model..

[73] Miller B.R., McGee T.D., Swails J.M., Homeyer N., Gohlke H., Roitberg A.E. (2012). MMPBSA.py: An Efficient Program for End-State Free Energy Calculations. J. Chem. Theory Comput..

[74] Sahakyan H. (2021). Improving virtual screening results with MM/GBSA and MM/PBSA rescoring. J. Comput. Mol. Des..

[75] Duan L., Liu X., Zhang J.Z. (2016). Interaction Entropy: A New Paradigm for Highly Efficient and Reliable Computation of Protein–Ligand Binding Free Energy. J. Am. Chem. Soc..

[76] Woods C.J., Malaisree M., Michel J., Long B., McIntosh-Smith S., Mulholland A.J. (2014). Rapid decomposition and visualisation of protein–ligand binding free energies by residue and by water. Faraday Discuss..

[77] Woods C.J., Malaisree M., Hannongbua S., Mulholland A.J. (2011). A water-swap reaction coordinate for the calculation of absolute protein–ligand binding free energies. J. Chem. Phys..

[78] Daina A., Michielin O., Zoete V. (2017). SwissADME: A free web tool to evaluate pharmacokinetics, drug-likeness and medicinal chemistry friendliness of small molecules. Sci. Rep..

