# Supplementary material for: Targeting Toxoplasma gondii ME49 TgAPN2: A Bioinformatics Approach for Antiparasitic Drug Discovery
Source: Molecules. 2023 Apr 3;28(7):3186. doi: 10.3390/molecules28073186 (PMC10096047; doi:10.3390/molecules28073186)
Supplement: Supplementary file 1 [file molecules-28-03186-s001.zip › molecules-2299046-supplementary.pdf]

Targeting *Toxoplasma gondii* ME49 TgAPN2: A Bioinformatics Approach for Antiparasitic Drug Discovery

Ali Altharawi

Department of Pharmaceutical Chemistry, College of Pharmacy, Prince Sattam Bin Abdulaziz University, Al-Kharj 11942, Saudi Arabia; a.altharawi@psau.edu.sa

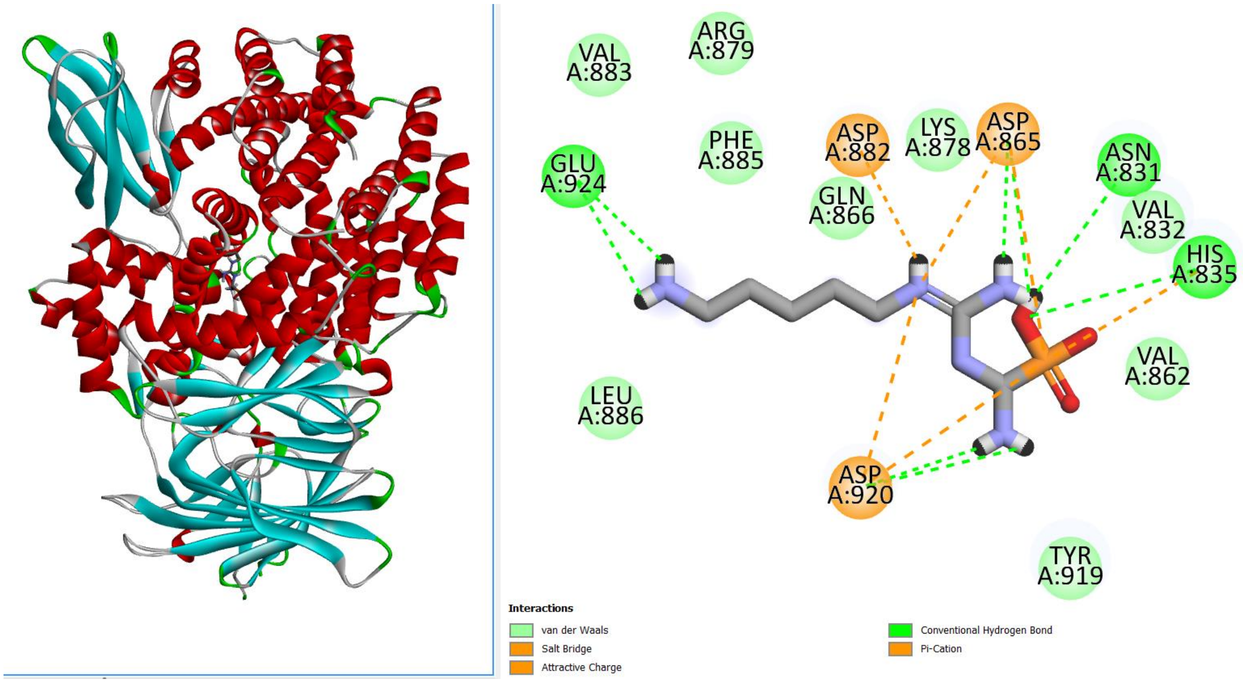

Figure S1. Docked conformation and interactions of control compound 1 with the TgAPN2 (show by secondary structure cartoon representation)

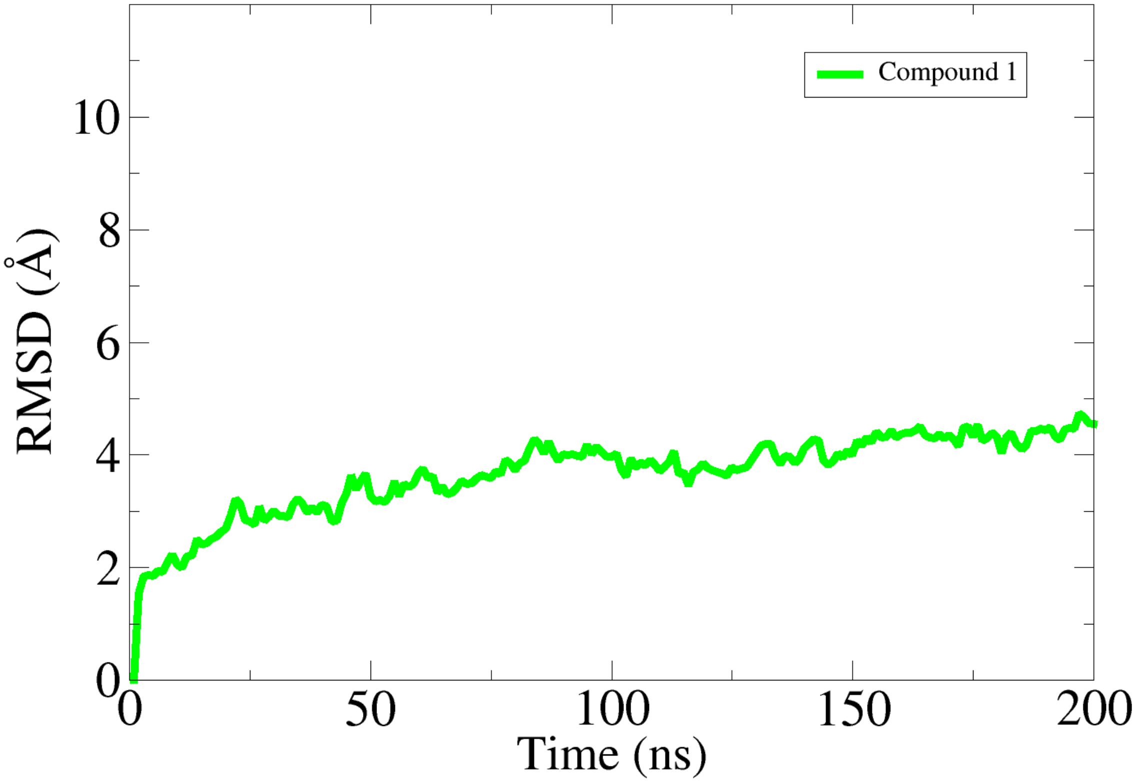

Figure S2. RMSD of control compound 1.
